# Looking for a bright side: Tales from a recreational therapy virtual service-learning project during a pandemic

**DOI:** 10.1093/geroni/igab046.2733

**Published:** 2021-12-17

**Authors:** Alyssa Doughty, Jennifer Taylor

**Affiliations:** 1 The Neighbors of Dunn County; 2 University of Wisconsin-La Crosse, La Crosse, Wisconsin, United States

## Abstract

The everchanging policies and inability to utilize university students due to COVID-19 impacted both residents living in long-term care as well as the next generation of students pursuing careers in the field. University Wisconsin-La Crosse (UWL) faculty strategized solutions as restrictions threatened to impact hands-on opportunities for students. Was there a safe and effective solution to offer residents evidence-based programming while also providing students with vital field experience? Simply stated, the answer was yes. Thus, the UWL Happiness Project was born. This session will outline the UWL Happiness Project, a ten-week, telehealth program implemented between a skilled nursing facility in rural Wisconsin and the UWL Therapeutic Recreation Program, an AGHE Program of Merit for Health Professions designated program. The evidence-based curriculum was developed by an emerging UWL graduate student scholar with faculty mentorship. The innovative curriculum focuses on increasing feelings of happiness using PERMA, a theoretical model grounded in positive psychology. During virtual sessions, older adult residents (ages 65-85) and students built connection while working through weekly focus areas (e.g. vitality, mindfulness, friendship). An overview of AGHE competencies addressed within the project, online course demonstration, and assignment development will be discussed along with information about how these connections fostered an opportunity for students to see aging from a different perspective. This is the first time we are presenting results from the newly developed program. In this, we look forward to sharing student measurements and outcomes, as well as lessons learned during this meaningful, stimulating, and insightful educational session.

